# Adult Tobacco Use Among Racial and Ethnic Groups Living in the United States, 2002–2005

**Published:** 2008-06-15

**Authors:** Ralph S Caraballo, Sue Lin Yee, Joe Gfroerer, Sara A Mirza

**Affiliations:** Office on Smoking and Health, National Center for Chronic Disease Prevention and Health Promotion, Centers for Disease Control and Prevention; Coordinating Center for Terrorism Preparedness and Emergency Response, Centers for Disease Control and Prevention, Atlanta, Georgia; Office of Applied Studies, Substance Abuse and Mental Health Services Administration, Rockville, Maryland; Office on Smoking and Health, National Center for Chronic Disease Prevention and Health Promotion, Centers for Disease Control and Prevention, Atlanta, Georgia

## Abstract

**Introduction:**

U.S. data on adult tobacco use and the relationship between such use and tobacco-related health disparities are primarily limited to broad racial or ethnic populations. To monitor progress in tobacco control among adults living in the United States, we present information on tobacco use for both aggregated and disaggregated racial and ethnic subgroups.

**Methods:**

We used data from the nationally representative sample of adults aged 18 years or older who participated in the National Survey on Drug Use and Health conducted 4 times during 2002–2005. We calculated 2 outcome measures: 1) use of any tobacco product (cigarettes, chewing or snuff tobacco, cigars, or pipes) during the 30 days before each survey and 2) cigarette smoking during the 30 days before each survey.

**Results:**

The prevalence of tobacco use among adults aged 18 years or older varied widely across racial or ethnic groups or subgroups. Overall, about 3 of 10 adults living in the United States were tobacco users during the 30 days before being surveyed. The population groups or subgroups with a tobacco-use prevalence of 30% or higher were African Americans, American Indians or Alaska Natives, Native Hawaiians or other Pacific Islanders, Puerto Ricans, and whites.

**Conclusion:**

These results indicate that the prevalence of adult tobacco use is still high among several U.S. population groups or subgroups. Our results also support the need to design and evaluate interventions to prevent or control tobacco use that would reach distinct U.S. adult population groups or subgroups.

## Introduction

Because only limited data are available on population groups and subgroups with disproportionately high rates of tobacco use, researchers face challenges in developing interventions and securing resources to implement tobacco control programs. Since the release in 1998 of the Surgeon General's first report to focus on tobacco use among 4 U.S. racial/ethnic minority groups (African Americans, American Indians or Alaska Natives, Asian Americans or other Pacific Islanders, and Hispanics) (1), researchers have collected tobacco-related information on specific U.S. population subgroups. However, many of these data are on population subgroups in states or communities rather than in the United States as a whole (2-11). Having state and local data is important because tobacco control interventions occur in states or communities, and many population groups and subgroups are concentrated in certain states or counties. However, having data for the entire United States is also important. The problem is that national data are often aggregated, which can mask important variations within population subgroups.

To monitor progress in tobacco control among racial/ethnic groups and subgroups of adults aged 18 years or older living in the United States, we analyzed self-reported data on tobacco use and cigarette smoking from 6 major racial or ethnic U.S. populations (African Americans, American Indians or Alaska Natives, Asians, Hawaiians or other Pacific Islanders, Hispanics, and whites), 6 Asian subpopulations (Chinese, Filipino, Asian Indian, Japanese, Korean, and Vietnamese), and 4 Hispanic subpopulations (Central or South American, Cuban, Mexican, and Puerto Rican). The data were also analyzed by sex. The racial and ethnic classifications used in this study adhere to the Office of Management and Budget's standards for collecting statistical data on race and ethnicity (12).

## Methods

### Data source

The National Survey on Drug Use and Health (NSDUH) (13) is a nationwide household survey that collects data on drug use and drug abuse, including tobacco use, from a representative sample of the U.S. civilian, noninstitutionalized population aged 12 years or older. Specifically, the NSDUH collects data on overall tobacco use, cigarette smoking, and other behavioral information related to cigarette smoking and brand preference. NSDUH data are collected through a computerized questionnaire administered in the privacy of participants' homes by a professional field interviewer who visits each selected household. Most responses are answered in private by the participant, although the interviewer reads and enters the responses to some questions in the presence of the participant. Questions about tobacco use were administered through audio, computer-assisted, self-interview methods to maximize privacy and improve reporting of sensitive behaviors. For this analysis, we combined data for adults aged 18 years or older from the 4 surveys conducted in 2002, 2003, 2004, and 2005 in order to obtain a sample size large enough to examine tobacco use and cigarette smoking within both aggregated and disaggregated racial or ethnic groups or subgroups.

### Study population

We included data on 2002–2005 NSDUH participants aged 18 years or older (N = 180,833) in our calculations of prevalence of cigarette use and tobacco use (Table 1). The average-weighted overall response rate for respondents aged 18 years or older in the 2002–2005 NSDUH surveys was 69.0%. This rate is the product of the weighted household screening response rate (90.9%) and the weighted response rate of individuals in each selected household (75.9%) during 2002–2005.

### Demographic classification

Race/ethnicity designation is based on respondents' self-classification. For Hispanic origin, respondents were asked, "Are you of Hispanic, Latino, or Spanish origin or descent?" Hispanics were also asked to select the specific subgroup (Mexican, Puerto Rican, Central or South American, or Cuban) that best described them. For race, respondents were asked, "Which of these groups best describes you?"  Response selections were white, black/African American, American Indian or Alaska Native, Native Hawaiian, other Pacific Islander, Asian, and other. Asians were also asked to select the subgroup (Chinese, Filipino, Japanese, Asian Indian, Korean, or Vietnamese) that best described them. Because of small sample size, the subgroups Hawaiian and Other Pacific Islanders were combined. For this study, all Hispanics are included in the Hispanic group regardless of race; all other race/ethnicity categories exclude Hispanics. We refer to non-Hispanic whites as whites and to non-Hispanic blacks as African Americans.

### Tobacco-related variables

The tobacco portion of NSDUH contains 43 items about the use of cigarettes, chewing tobacco, snuff (i.e., dip), cigars, or pipes. A cigarette smoker is defined as anyone who answered "yes" to the question "During the past 30 days, have you smoked part or all of a cigarette?" Anyone who answered "yes" to either the cigarette question or to a similar question about each type of tobacco product was considered to be a current tobacco user.

### Statistical analysis

We cross-tabulated the outcome variables of interest by race and ethnicity. Data on individuals identifying themselves as being of multiple races were included in the aggregated data but were not included in the data for a racial or ethnic subgroup. Confidence intervals (95%) were calculated for all point estimates. We used *t* tests to determine any significant differences between men and women in each ethnic or racial group or subgroup. We also used *t *tests to compare estimates for each racial or ethnic group with the estimates for the overall U.S. total (for men, for women, and for both sexes combined). All prevalence measures and confidence intervals were estimated using SAS SUDAAN (RTI International, Research Triangle Park, North Carolina). Survey weights were used to account for different probabilities of selection.

## Results

### Prevalence of U.S. tobacco use

Overall, we found substantial differences among racial or ethnic groups and subgroups in the prevalence of tobacco use during the 30 days before each survey: it ranged from 42.6% for American Indians or Alaska Natives to 10.0% for Chinese (Table 2). Using the total prevalence of tobacco use among U.S. adults (31.5%) as the referent group, the data indicate that whites (33.0%) and American Indians or Alaska Natives (42.6%) had a significantly higher prevalence of tobacco use than did the total of U.S. adults (Table 2). African Americans, Hawaiians or other Pacific Islanders, Koreans, Puerto Ricans, and Cubans had statistically similar prevalences of tobacco use. Chinese, Filipinos, Japanese, Asian Indians, Vietnamese, Mexicans, and Central or South Americans had lower prevalences of tobacco use than did the total of U.S. adults.

Among men, whites (40.0%) and American Indians or Alaska Natives (48.2%) had significantly higher prevalences of tobacco use than did the total of U.S. men (38.7%). African Americans, Hawaiians or other Pacific Islanders, Koreans, Vietnamese, Puerto Ricans, and Cubans had statistically similar prevalences of tobacco use to that of U.S. men in general. Chinese, Filipinos, Japanese, Asian Indians, Mexicans, and Central or South Americans had lower prevalences of tobacco use than did the total of U.S. men.

Among women, whites (26.6%) and American Indians or Alaska Natives (37.9%) had higher prevalences of tobacco use than did the total of U.S. adult women (24.9%). African Americans, Hawaiians or other Pacific Islanders, Koreans, Puerto Ricans, and Cubans had statistically similar prevalences of tobacco use to the prevalence of U.S. women in general. Chinese, Filipinos, Japanese, Asian Indians, Vietnamese, Mexicans, and Central or South Americans had lower prevalences of tobacco use than did the total of U.S. women.

In all racial or ethnic groups and subgroups, men had significantly higher prevalences of tobacco use than did women. For some subgroups (e.g., many Asian subgroups), the difference in tobacco use between men and women was substantial.

### Prevalence of U.S. cigarette use

The overall prevalence of cigarette smoking among U.S. adults during the 30 days before being surveyed was 26.9% (Table 3). Whites (27.7%), American Indians or Alaska Natives (37.1%), and Puerto Ricans (31.5%) had significantly higher prevalences of smoking than did the total of U.S. adults (Table 3). African Americans, Hawaiians or other Pacific Islanders, Koreans, Vietnamese, and Cubans had statistically similar prevalences of cigarette smoking to that of the total of U.S. adults. Chinese, Filipinos, Japanese, Asian Indians, Mexicans, and Central or South Americans had lower prevalences of smoking than did the total of U.S. adults.

Among men, African Americans (33.6%), American Indians or Alaska Natives (39.3%), and Puerto Ricans (35.6%) had significantly higher prevalences of cigarette smoking than did the total of U.S. men (30.0%). Whites, Hawaiians or other Pacific Islanders, Filipinos, Koreans, Vietnamese, Mexicans, and Cubans had statistically similar prevalences of cigarette smoking to the prevalence of the total of U.S. men. Chinese, Japanese, Asian Indian, and Central or South American men had prevalences of smoking significantly lower than the prevalence of the total of U.S. men.

Among women, whites (25.9%) and American Indians or Alaska Natives (35.2%) had significantly higher prevalences of cigarette smoking than did the total of U.S. women (23.9%). African Americans, Hawaiians or other Pacific Islanders, Koreans, Puerto Ricans, and Cubans had statistically similar prevalences of cigarette smoking to the prevalence of the total of U.S. women (Table 3). Chinese, Filipino, Japanese, Asian Indian, Vietnamese, Mexican, and Central or South American women had lower prevalences of smoking than did the total of U.S. women.

For all groups, prevalence estimates of cigarette smoking were higher for men than for women, but the differences were not statistically significant for American Indians or Alaska Natives, Hawaiians or other Pacific Islanders, and Cubans.

An examination of the tobacco use prevalences in Table 2 and cigarette smoking prevalences in Table 3 shows that, for some population subgroups, the difference between overall tobacco use and cigarette use only is several percentage points. For example, overall, about 14.6% of tobacco users in the United States did not smoke cigarettes (31.5% tobacco users vs 26.9% cigarette smokers) (calculation not shown). A larger percentage of male tobacco users (22.5%) than female tobacco users (4.0%) were not cigarette smokers. Specifically, we found wide differences in this indicator between men and women within these racial or ethnic groups: whites (25.7% vs 2.6%), Native Hawaiians or other Pacific Islanders (14.3% vs 1.5%), Japanese (28.3% vs 1.2%), Koreans (10.3% vs 1.5%), Puerto Ricans (11.2% vs 2.1%), and Cubans (17.9% vs 5.3%) (results not shown in tables).

The prevalences obtained through the 2002–2005 NSDUH surveys are higher (5.4% overall, 5.5% for men, 3.1% for women, 5.1% for whites, 6.2% for African Americans, 2.1% for Asians, 7.8% for Hispanics) than results obtained through the National Health Interview Survey (NHIS), which is also conducted with the adult U.S. noninstitutionalized civilian population (14). Results for American Indians or Alaska Natives are similar for both surveys.

## Discussion

The findings of this study indicate broad disparities in both tobacco use and cigarette smoking by race or ethnicity; widespread differences by sex were also noted. Our results challenge the belief among some public health practitioners that Asians and Hispanics have a low prevalence of tobacco or cigarette use (15,16).

In addition, we found that some population groups or subgroups are far from reaching the *Healthy People 2010* (HP 2010) objective for tobacco use or cigarette smoking (≤12%), whereas other groups have already achieved this goal (17). Although no group or subgroup of men had reached the *HP 2010* goal of 12% or less for cigarette smoking, 5 subgroups of Asian women (Chinese, Filipino, Japanese, Asian Indian, and Vietnamese) have achieved this goal. It is possible, however, that these Asian subgroups of women never had a smoking prevalence as high as 12%. It is important to learn how prevalence estimates for men can be reduced at least to the levels for women in the same racial or ethnic group (18-29).

In 2005, cigarette companies spent $13.11 billion on advertising and promotional expenses, down from $15.12 billion in 2003, but nearly double what was spent in 1998 (30). From 2002 to 2006, spending by state programs to control tobacco use declined from $749.7 million to $551.0 million, an amount less than 3% of the $21.3 billion that the states received in 2005 from tobacco-excise taxes and the 1998 Tobacco Master Settlement Agreement (30). Certain tobacco products are advertised and promoted disproportionately to members of minority racial communities (1). For example, marketing to Hispanics and American Indians or Alaska Natives often includes advertising and promoting cigarette brands with names such as Rio, Dorado, and American Spirit, and the tobacco industry has sponsored Tet festivals and activities related to Asian American Heritage Month (1). Research findings suggest that three African American publications — *Ebony, Jet,* and *Essence* — receive higher revenues from tobacco companies than do mainstream publications (1). Implementing tobacco control programs that reach specific racial or ethnic groups living in the United States with culturally appropriate interventions might reduce tobacco use and cigarette smoking among members of those groups. Comprehensive approaches that use culturally appropriate, targeted media and education campaigns and that increase the capacity of racial or ethnic populations to address tobacco use within their communities have been advocated (31). The systematic reviews in *Guide to Community Preventive Services* (32) of the effectiveness of many interventions to reduce or prevent tobacco use may help many racial or ethnic groups and subgroups to develop tailored tobacco-control programs. However, many interventions were designed on the basis of studies of the predominantly white population, so it is not clear whether the same interventions would be effective with other groups or subgroups.

The difference in prevalences obtained through the 2002–2005 NSDUH surveys and the NHIS could be accounted for through differences in sampling methods, protocols of participant contact, methods of data collection, instrumentation, analytic methodology, or chance. The difference in how each survey is administered is important and has been shown to affect respondents' reporting of tobacco use (33). Specifically, the tobacco questions are self-administered in the NSDUH and interviewer-administered in the NHIS. An experiment embedded in the 1994 NSDUH found that significantly higher rates of adult cigarette use were reported with self-administration (33). In addition, in 2002 NSDUH introduced design changes that included the offer of a cash incentive to enhance the likelihood of participation and an improvement on the accuracy of the tobacco-related self-reported information (i.e., a reduction in false negatives). These changes may have resulted in findings of increases in prevalence. The magnitude of the effect on the survey was sufficient for NSDUH to consider 2002 data to be a new baseline for measuring trends.

Our study has at least 2 limitations. First, respondents were able to complete the interviews only in English or Spanish. The absence of an option to respond in another language (e.g., Mandarin, Korean, Hindi) may have contributed to inaccurate estimates of tobacco or cigarette use among some subgroups. Second, separate data are presented for Asian and Hispanic subgroups but not for other subgroups (e.g., not for individual American Indian tribes or African American subgroups).

Many chronic diseases (e.g., cardiovascular disease, lung disease, and many cancers)Many chronic diseases (e.g., cardiovascular disease, lung disease, and many cancers) are caused by cigarette smoking and other tobacco use. If we are to reduce the prevalence of these diseases, it is critical to prevent or reduce tobacco use among all racial or ethnic groups and subgroups and to reduce the racial disparities in the burden of tobacco-related disease. Sustaining strong local and state comprehensive tobacco control programs is essential if we are to succeed in 1) decreasing tobacco use by racial and ethnic groups and subgroups with high smoking prevalences and 2) preventing increases in tobacco use by racial and ethnic groups and subgroups that have low prevalences of tobacco use. We need to focus our efforts on launching effective and culturally competent interventions and on strengthening policies that control tobacco use (e.g., smoke-free environments, high prices for tobacco products, health insurance coverage for programs to help people stop using tobacco) within racial and ethnic communities with high prevalences of tobacco use. By investing in programs that address the individual needs of diverse populations, we can make tremendous progress in eliminating the disparities in tobacco use and tobacco-related diseases.

## Figures and Tables

**Table 1:** Number of Survey Respondents Aged 18 or Older, by Race or Ethnicity and Sex, National Survey on Drug Use and Health, 2002–2005

Race or Ethnicity	Sex		Total^a^

	Male	Female	
Total Study Subjects^a^	84,429	96,404	180,833
**Non-Hispanic**			
Total Non-Hispanic^a^	72,966	84,143	157,109
White	58,714	65,691	124,405
African American	8,508	11,938	20,446
American Indian or Alaska Native	1,035	1,188	2,223
Native Hawaiian or other Pacific Islander	376	359	735
**Asian^a^**			
Total Asian^a^	2,741	3,012	5,753
Chinese	532	585	1,117
Filipino	486	621	1,107
Japanese	270	341	611
Asian Indian	707	669	1,376
Korean	227	315	542
Vietnamese	254	197	451
**Hispanic**			
Total Hispanic^a^	11,463	12,261	23,724
Mexican	7,091	7,151	14,242
Puerto Rican	1,237	1,571	2,808
Central or South American	1,987	2,052	4,039
Cuban	394	451	845

a Totals include respondents who reported racial/ethnic subgroups not shown and respondents who reported being from more than 1 racial or ethnic subgroup.

**Table 2:** Percentage of Respondents Aged 18 or Older Who Used Tobacco Products^a^ During the 30 Days Before Being Surveyed, by Race or Ethnicity and Sex, National Survey on Drug Use and Health, 2002–2005

Race or Ethnicity	Sex		Total^b^ (95% CI)

	Male (95% CI)	Female (95% CI)	
Total Study Subjects^b^	38.7^c^ (38.1-39.2)	24.9^c^ (24.4-25.3)	31.5 (31.1-31.9)
**Non-Hispanic**			
Total Non-Hispanic^b^	39.4^c^^,^ d (38.8-40.0)	25.7^c^^,^ d (25.2-26.2)	32.2^d^ (31.8-32.6)
White	40.0^c^^,^ d (39.3-40.7)	26.6^c^^,^ d (26.0-27.1)	33.0^d^ (32.6-33.5)
African American	39.8^c^ (38.0-41.5)	25.4^c^ (24.1-26.7)	31.8 (30.7-32.9)
American Indian or Alaska Native	48.2^d^^,^ e (41.1-55.4)	37.9^d^^,^ e (32.7-43.3)	42.6^d^ (38.3-47.0)
Native Hawaiian or other Pacific Islander	41.9^e^ (32.1-52.4)	27.0^e^ (20.3-34.9)	34.6 (28.4-41.5)
**Asian**			
Total Asian^b^	24.0^c^^,^ d (21.4-26.7)	8.4^c^^,^ d (7.1-9.9)	15.8^d^ (14.3-17.3)
Chinese	16.1^c^^,^ d (12.1-21.1)	4.9^c^^,^ d (3.1-7.8)	10.0^d^ (7.8-12.8)
Filipino	26.0^c^^,^ d (20.0-33.0)	10.4^c^^,^ d (7.3-14.5)	17.0^d^ (13.9-20.5)
Japanese	24.0^c^^,^ d (16.7-33.3)	8.1^c^^,^ d (5.3-12.2)	15.2^d^ (11.5-19.9)
Asian Indian	20.7^c^^,^ d (15.9-26.6)	3.6^c^^,^ d (2.5-5.3)	12.8^d^ (10.0-16.4)
Korean	41.7^c^ (32.0-52.0)	20.4^c^ (14.3-28.1)	28.4 (22.9-34.6)
Vietnamese	33.5^c^ (25.5-42.6)	8.9^c^ (5.1-15.1)	22.5^d^ (17.3-28.7)
**Hispanic**			
Total Hispanic^b^	34.0^c^^,^ d (32.5-35.5)	18.2^c^^,^ d (17.0-19.4)	26.3^d^ (25.3-27.3)
Mexican	34.8^c^^,^ d (33.0-36.7)	16.4^c^^,^ d (15.0-17.8)	26.1^d^ (24.9-27.3)
Puerto Rican	40.1^c^ (34.9-45.4)	28.6^c^ (24.5-33.1)	33.9 (30.5-37.6)
Central or South American	27.3^c^^,^ d (23.8-31.1)	15.6^c^^,^ d (12.7-19.0)	21.6^d^ (19.4-24.1)
Cuban	35.7^c^ (29.2-42.6)	22.7^c^ (16.7-30.1)	28.9 (24.5-33.8)

CI indicates confidence interval.

a Tobacco products include cigarettes, smokeless tobacco (i.e., chewing tobacco or snuff), cigars, and pipe tobacco.

b Total includes data on respondents who reported being of racial or ethnic subgroups not shown in table and respondents who reported being of more than 1 subgroup.

c Difference between the estimates for men and women in the same racial or ethnic group is statistically significant at the 0.01 level: *t* test.

d Difference between this estimate and the estimate for the overall total (top row, same column) is statistically significant at the 0.01 level: *t* test.

e Difference between the estimates for men and women in the same racial or ethnic group is statistically significant at the 0.05 level: *t* test.

**Table 3:** Percentage of Respondents Aged 18 or Older Who Smoked Cigarettes During the 30 Days Before Being Surveyed, by Race or Ethnicity and Sex, National Survey on Drug Use and Health, 2002–2005

Race or Ethnicity	Sex		Total^a^ (95% CI)

	Male (95% CI)	Female (95% CI)	
Total Study Subjects^a^	30.0^b^ (29.5-30.5)	23.9^b^ (23.5-24.4)	26.9 (26.5-27.2)
**Non-Hispanic**			
**Total non-Hispanic^a^**	30.0^b^ (29.5-30.5)	24.8^b^^,^ c (24.3-25.3)	27.3^c^ (26.9-27.7)
White	29.7^b^ (29.1-30.3)	25.9^b^^,^ c (25.3-26.4)	27.7^c^ (27.3-28.2)
African American	33.6^b^^,^ c (32.0-35.3)	22.8^b^ (21.6-24.1)	27.6 (26.6-28.7)
American Indian or Alaska Native	39.3^c^ (32.9-46.1)	35.2^c^ (30.0-40.8)	37.1^c^ (32.9-41.4)
Native Hawaiian or Other Pacific Islander	35.9 (26.8-46.0)	26.6 (20.0-34.5)	31.4 (25.4-38.0)
**Asian**			
Total Asian^a^	21.6^b^^,^ c (19.2-24.2)	8.1^b^^,^ c (6.8-9.6)	14.5^c^ (13.1-16.0)
Chinese	13.9^b^^,^ c (10.4-18.3)	4.6^b^^,^ c (2.8-7.4)	8.8^c^ (6.9-11.3)
Filipino	25.5^b^ (19.5-32.5)	10.2^b^^,^ c (7.2-14.4)	16.7^c^ (13.7-20.2)
Japanese	17.2^c^^,^ d (11.7-24.6)	8.0^c^^,^ d (5.1-12.1)	12.1^c^ (9.2-15.8)
Asian Indian	19.1^b^^,^ c (14.3-24.9)	3.5^b^^,^ c (2.4-5.2)	11.9^c^ (9.1-15.4)
Korean	37.4^b^ (28.2-47.6)	20.1^b^ (14.1-27.8)	26.6 (21.3-32.7)
Vietnamese	32.5^b^ (24.6-41.5)	8.0^b^^,^ c (4.4-14.0)	21.5 (16.4-27.7)
**Hispanic**			
**Total Hispanic^a^**	30.1^b^ (28.6-31.6)	17.5^b^^,^ c (16.3-18.7)	23.9^c^ (23.0-24.9)
Mexican	31.0^b^ (29.2-32.8)	15.7^b^^,^ c (14.4-17.2)	23.8^c^ (22.6-24.9)
Puerto Rican	35.6^d^^,^ e (30.2-41.3)	28.0^d^ (23.9-32.5)	31.5^e^ (28.0-35.2)
Central or South American	25.3^b^^,^ c(21.9-29.1)	14.7^b^^,^ c(11.9-18.0)	20.2^c^ (18.0-22.6)
Cuban	29.3 (23.3-36.0)	21.5 (15.6-28.9)	25.2 (21.0-30.0)

CI indicates confidence interval.

a Totals include data on respondents who reported being of racial or ethnic subgroups not shown and on respondents who reported being of more than 1 racial or ethnic group.

b Difference between estimates for men and women in the same racial/ethnic group is statistically significant at the 0.01 level: *t *test.

c Difference between this estimate and the estimate for the overall total (top row, same column) is statistically significant at the 0.01 level:* t* test.

d Difference between estimates for men and women in the same racial/ethnic group is statistically significant at the 0.05 level: *t* test.

e Difference between this estimate and the estimate for all Hispanics is statistically significant at the 0.05 level: *t* test.
